# “I Am a Total…Loser” – The Role of Interpretation Biases in Youth Depression

**DOI:** 10.1007/s10802-020-00670-3

**Published:** 2020-07-11

**Authors:** Anca Sfärlea, Christina Buhl, Johanna Loechner, Jakob Neumüller, Laura Asperud Thomsen, Kornelija Starman, Elske Salemink, Gerd Schulte-Körne, Belinda Platt

**Affiliations:** 1 Department of Child and Adolescent Psychiatry, Psychosomatics and Psychotherapy, University Hospital, LMU Munich, Nußbaumstr. 5a, 80336 Munich, Germany; 2 Department of Clinical Psychology and Psychotherapy, LMU Munich, Munich, Germany; 3 Department of Clinical Psychology, Utrecht University, Utrecht, The Netherlands

**Keywords:** Interpretation bias, Major depression, Children and adolescents, Familial risk for depression, Ambiguous scenarios task, Scrambled sentences task

## Abstract

Negative interpretation biases have been found to characterize adults with depression and to be involved in the development and maintenance of the disorder. However, less is known about their role in youth depression. The present study investigated i) whether negative interpretation biases characterize children and adolescents with depression and ii) to what extent these biases are more pronounced in currently depressed youth compared to youth at risk for depression (as some negative interpretation biases have been found already in high-risk youth before disorder onset). After a negative mood induction interpretation biases were assessed with two experimental tasks: Ambiguous Scenarios Task (AST) and Scrambled Sentences Task (SST) in three groups of 9–14-year-olds: children and adolescents with a diagnosis of major depression (*n* = 32), children and adolescents with a high risk for depression (children of depressed parents; *n* = 48), as well as low-risk children and adolescents (*n* = 42). Depressed youth exhibited substantially more negative interpretation biases than both high-risk and low-risk groups (as assessed with both tasks), while the high-risk group showed more negative interpretation biases than the low-risk group only as assessed via the SST. The results indicate that the negative interpretation biases that are to some extent already present in high-risk populations before disorder onset are strongly amplified in currently depressed youth. The different findings for the two tasks suggest that more implicit interpretation biases (assessed with the SST) might represent cognitive vulnerabilities for depression whereas more explicit interpretation biases (assessed with the AST) may arise as a consequence of depressive symptomatology.

**Electronic supplementary material:**

The online version of this article (10.1007/s10802-020-00670-3) contains supplementary material, which is available to authorized users.

## Introduction

Depression is one of the most common psychiatric disorders in childhood and adolescence (Costello et al. 2003; Lewinsohn et al. 1993) with up to 20% of young people having experienced at least one episode of major depression (MD) by the end of adolescence (Thapar et al. 2012). Early-onset MD is associated with adverse outcomes later in life such as educational underachievement (Fergusson and Woodward 2002), impairments in psychosocial functioning (Hammen et al. 2008), and reduced life satisfaction (Lewinsohn et al. 2003). In addition, early-onset MD often follows a recurrent course (e.g., Lewinsohn et al. 1999; Weissman et al. 1999), which further contributes to the negative consequences of the disorder (Wilson et al. 2015; Hammen et al. 2008).

Cognitive theories of depression propose that cognitive vulnerabilities such as cognitive biases play a crucial role in the development and maintenance of depressive disorders (e.g., A. T. Beck and Haigh 2014; Disner et al. 2011). Negative cognitive biases are tendencies to preferentially process negative compared to positive or neutral information and can be found on various levels of information processing, including attention, interpretation, and memory (Everaert et al. 2012; LeMoult and Gotlib 2019). Negative interpretation biases, in particular, refer to tendencies to create more negative and fewer positive meanings to explain ambiguous emotional information (Everaert et al. 2017). For example, a situation in which one is giving a speech in front of a group and people are laughing could be interpreted negatively in terms of people laughing at one or positively in terms of people appreciating one’s jokes. In adults, the association between negative interpretation biases and depression has received particularly substantial empirical support (see Everaert et al. 2017, for a comprehensive meta-analysis).

However, results obtained from studies on adults with MD cannot be directly transferred onto depressed youth (Lakdawalla et al. 2007), as major cognitive and affective development is ongoing during childhood and adolescence (Blakemore and Choudhury 2006; Steinberg 2005). Therefore, cognitive vulnerabilities might either play a smaller role in youth than adult depression as cognitive patterns might not have evolved into stable, trait-like “cognitive styles” yet at this younger age (e.g., Lakdawalla et al. 2007). Alternatively, young people might be particulartly susceptible to negative cues in ambiguous emotional information due to brain maturation and hormonal changes associated with an enhanced emotional sensitivity (see e.g., Paus et al. 2008), resulting in more pronounced negative cognitive biases. Considering the particularly detrimental consequences of early-onset MD, understanding the mechanisms that are involved in the development and maintenance of the disorder at this early age is crucial in order to improve prevention and early intervention (Loechner et al. 2018; Weisz et al. 2006).

Still, research on the association of interpretation biases and depression in children and adolescents is rather scarce (Platt et al. 2017). Some studies have reported correlations between interpretation bias scores and depressive symptoms in unselected adolescent samples (e.g., Klein et al. 2018; Orchard et al. 2016a; Smith et al. 2018) as well as samples with elevated symptoms of depression (de Voogd et al. 2017), but only two studies have compared interpretation biases in clinically depressed versus healthy youth. As part of a validity check in their study of an intervention for clinically depressed adolescents and young adults (14–21 years old), Micco et al. (2014) compared the depressed group’s baseline interpretation bias (assessed with the experimental Ambiguous Scenarios Task, AST; Mathews and Mackintosh 2000) with that of a healthy control group and found the depressed adolescents and young adults to show a more negative interpretation bias. However, as the comparison of depressed and non-depressed groups was not the main aim of the study, this result is presented only briefly and its importance is not discussed. Orchard et al. (2016b) on the other hand, used the Ambiguous Scenarios Test for Depression in Adolescents, a questionnaire measure they had previously adapted and validated (Orchard et al. 2016a), to investigate interpretation biases in 12–18-year-old adolescents. They found a more negative interpretation bias in adolescents with a diagnosis of MD not only compared to healthy adolescents from the community but also to clinically-referred non-depressed youth and adolescents from the community with elevated depressive symptoms.

To date, no study has focused on comparing interpretation biases in depressed and non-depressed youth using experimental tasks. These do not rely on participants’ awareness of their depressive cognitions and are less prone to distortions due to demand characteristics (i.e., participants matching their responses to the experimenter’s presumed expectation), response biases (i.e., participants endorsing negative responses irrespective of the content corresponding to their interpretation or not), and deliberate response strategies (i.e., participants generating their responses based on a voluntary strategy instead of their immediate reaction to the ambiguous information) that are typical for self-report measures (e.g., Gotlib and Joormann 2010; Hirsch et al. 2016). Thus, experimental tasks enable a more objective assessment of cognitive processes and allow more automatic and unconscious processes that operate outside a person’s awareness to be captured. Therefore, the first aim of the present study was to investigate interpretation biases in youth depression using age-adapted experimental approaches to assess interpretation biases in children and adolescents with MD.

We administered the AST (Mathews and Mackintosh 2000) in which participants read several self-referent ambiguous scenarios and are then presented with different interpretations of each scenario. Interpretation bias is indexed by the difference between the endorsement of negative and positive interpretations (de Voogd et al. 2017). In addition, the Scrambled Sentences Task (SST; Wenzlaff and Bates 1998), which was specifically developed to assess interpretation biases in depressive disorders, was applied. In this task, participants form sentences out of arrays of words which can be either positive or negative. The proportion of negatively resolved sentences indicates the interpretation bias. Applying two experimental measures of interpretation bias allows the examination of different aspects of interpretation, with the AST presumably measuring a more conscious and explicit aspect and the SST capturing a more automatic and implicit aspect (Sfärlea et al. 2019). Both tasks have already been used in adolescent samples (e.g., de Voogd et al. 2017; Burnett Heyes et al. 2017) where they demonstrated at least acceptable reliability (Micco et al. 2014; Sfärlea et al. 2019).

Children and adolescents with MD were compared to two groups of non-depressed children and adolescents that varied in their risk for depression: children of parents with a history of depression, who are known to have an increased risk for MD themselves (e.g., Weissman et al. 2006) and children of parents with no history of depression or any other mental disorder, who have a low risk for depressive disorders. This allowed us to pursue the second aim of our study: to determine the extent to which interpretation biases are more pronounced in currently depressed youth compared to at-risk youth (that have been found to be characterized by more negative interpretation biases than youth at low risk for depression; Dearing and Gotlib 2009; Sfärlea et al. 2019). While negative interpretation biases in children and adolescents at high risk for depression indicate that these biases might be cognitive vulnerabilities or risk factors contributing to the development of depression (as suggested by theoretical models, e.g., Disner et al. 2011), even more pronounced interpretation biases in currently depressed children and adolescents indicate that these biases might be exacerbated as a consequence of depressive symptomatology. No study to date has directly compared interpretation biases in depressed, high-, and low-risk youth. One study that investigated memory biases in children and adolescents with MD, children and adolescents whose mothers were affected by MD, and children and adolescents without familial history of MD (Fattahi Asl et al. 2015) found negative memory biases in both depressed as well as at-risk youth compared to low-risk youth. However, the negative memory biases were more pronounced in currently depressed children and adolescents than in the at-risk group.

In order to be able to compare currently depressed youth to at-risk youth we focused on children and adolescents aged 9–14 years. Children younger than 9 years were not included due to concerns about their ability to understand and perform the tasks. Adolescents older than 14 years were not included since the incidence of depression in children of parents with a history of depression increases substantially after that age (e.g., Weissman et al. 2006), and investigating older children of depressed parents that had not yet suffered from an episode of MD might result in examining a particularly resilient and therefore non-representative high-risk sample.

With respect to the first aim of the study, we expected to find more negative interpretation biases in children and adolescents with MD in comparison to healthy children and adolescents (both high- and low-risk youth), based on theoretical predictions (e.g., Disner et al. 2011) and previous findings (Orchard et al. 2016b; Micco et al. 2014). Regarding the second aim, we expected negative interpretation biases to be to some extent present in youth at high risk for depression compared to youth at low risk for depression (corresponding to our previous results, Sfärlea et al. 2019; as well as Dearing and Gotlib 2009; Goodman and Gotlib 1999), but to be more pronounced in depressed versus high-risk youth (as found for memory biases; Fattahi Asl et al. 2015).

## Methods

The present data on interpretation biases were collected within a broader project on cognitive biases in depressed as well as high- and low-risk youth. It was planned as an add-on to a study on cognitive biases in the offspring of depressed versus non-depressed parents (Platt 2017; Sfärlea et al. 2019). Data from interpretation bias tasks^1^ are presented here while data from attention bias tasks are presented elsewhere (Buhl et al. in preparation; Platt et al. submitted).

### Participants

A total of 122 children and adolescents aged 9–14 years were included in the data analysis.^2^ The sample consisted of *n* = 32 children and adolescents with MD, *n* = 48 children and adolescents at high familial risk for depression (HR group), and *n* = 42 children and adolescents at low familial risk for depression (LR group). The data from 87% of the HR and LR children was collected within a study investigating the transgenerational transmission of cognitive biases (Platt 2017; Sfärlea et al. 2019), in which they participated with one of their parents. Of the HR children, 28 were recruited through a study evaluating an intervention to prevent the development of depression in children of parents with a history of depression (Platt et al. 2014). Eleven of those had already received the prevention program by the time they took part in the present study while the others took part before receiving the intervention. Other HR as well as the LR families were recruited via local advertisements, previous studies, and mailings to randomly-selected families with children in the corresponding age range provided by the local registry office. Youth with MD were mostly in- or outpatients from a Department of Child and Adolescent Psychiatry, Psychosomatics and Psychotherapy of the University Hospital of the LMU Munich, *n* = 2 were recruited through licensed outpatient psychotherapists, and *n* = 3 were respondents to our mailings.

Exclusion criteria for all participants were intelligence quotient (IQ) < 85^3^ (assessed with the CFT 20-R; Weiß 2006), pervasive developmental disorders, attention deficit and hyperactivity disorder, and a history of schizophrenia or bipolar disorder. Children and adolescents were included in the MD group if they currently met criteria for MD according to DSM-IV^4^ (American Psychiatric Association 2000) as assessed with a standardized psychiatric interview (see below). Of the 32 participants in this group, *n* = 4 had recurrent episodes of MD, *n* = 2 were partially remitted (analyses excluding these participants revealed the same pattern of results), *n* = 15 fulfilled criteria for at least one comorbid anxiety disorder, and *n* = 3 (9.4%) were receiving psychotropic medication (selective serotonin reuptake inhibitors). Children and adolescents were included in the HR group if they did not meet criteria for any current or past axis I disorder^5^ but at least one of their parents met criteria for MD (*n* = 46) or dysthymia (*n* = 2; analyses excluding these participants revealed the same pattern of results) during the child’s lifetime. Children of parents with a history of bipolar disorder, schizophrenia, or substance abuse were not included. Children and adolescents were included in the LR group if they did not meet criteria for any current or past axis I disorder and none of their parents met criteria for any past or current axis I disorder.

All procedures were approved by the ethics committee of the Medical Faculty of the LMU Munich (Project 441–15). Written informed consent was obtained from all participants and their parents after a comprehensive explanation of the study procedures. HR and LR participants who participated together with their parents in the study on transgenerational transmission of cognitive biases received a reimbursement of 50 € per family while participants taking part only in this study received a reimbursement of 30 €.

### Psychopathology Assessment

All participants underwent extensive diagnostic assessment before inclusion in the study. A standardized, semi-structured psychiatric interview (K-DIPS; Schneider et al. 2009) was conducted with both participants and one of their parents to assess psychiatric diagnoses in children and adolescents. The K-DIPS is a well-established German diagnostic interview that allows diagnosis of a wide range of psychiatric axis I disorders according to DSM-IV (American Psychiatric Association 2000) with good interrater-reliability (accordance rates of at least 97% were reported for all diagnoses; Neuschwander et al. 2013). The interviews were conducted and evaluated by trained interviewers. Interrater-reliability was determined for 18% of the participants of the HR and LR groups by an independent researcher re-rating audio recordings of the diagnostic interviews and found the accordance rate for lifetime diagnosis of depression (pre-defined criterion) to be 100%. Interviews from the MD group were not audiotaped, but the participants in this group were referred to our study because they had a clinical diagnosis of depression which was confirmed with the diagnostic interview.

The adult version of the interview (DIPS; Schneider and Margraf 2011) was used to assess psychiatric diagnoses in the parents of the HR and LR participants (for HR participants it was applied to the parent affected by depression; for LR participants it was applied to both parents whenever possible, i.e., for 79% of participants). Interrater-reliability of the DIPS has been found to be good (with accordance rates of at least 87% reported for all diagnoses; Suppiger et al. 2008) and the accordance rate for lifetime diagnosis of depression was 94% in our sample. In addition, depressive symptoms of the parents were assessed with the German version of the Beck Depression Inventory-II (BDI-II; Hautzinger et al. 2006, obtained from both parents for 81% of HR and LR participants) and differed significantly (*t*s = 3.2, *p*s ≤ .002) between parents of HR (parent with a history of MD: *M* = 9.9, *SD* = 8.5; other parent: *M* = 4.2, *SD* = 4.5) and LR participants (*M* = 1.6, *SD* = 3.2).

Depressive symptoms of the participants were assessed with the German version of the Children’s Depression Inventory (DIKJ; Stiensmeier-Pelster et al. 2014) and anxiety symptoms were measured by the trait scale of the German version of the State Trait Anxiety Inventory for Children (STAIC; Unnewehr et al. 1992). A score for depressive symptoms was available for 121 and a score for anxiety symptoms for 117 of the 122 participants. Reliability of both self-report measures was excellent in our sample (DIKJ: Cronbach’s α = .96; STAIC-T: Cronbach’s α = .93).

### Ambiguous Scenarios Task

A computerized version of the AST (Mathews and Mackintosh 2000; adapted from Belli and Lau 2014) was used to assess the tendency to interpret ambiguous situations as positive or negative.

#### Stimuli

**Fig. 1:**
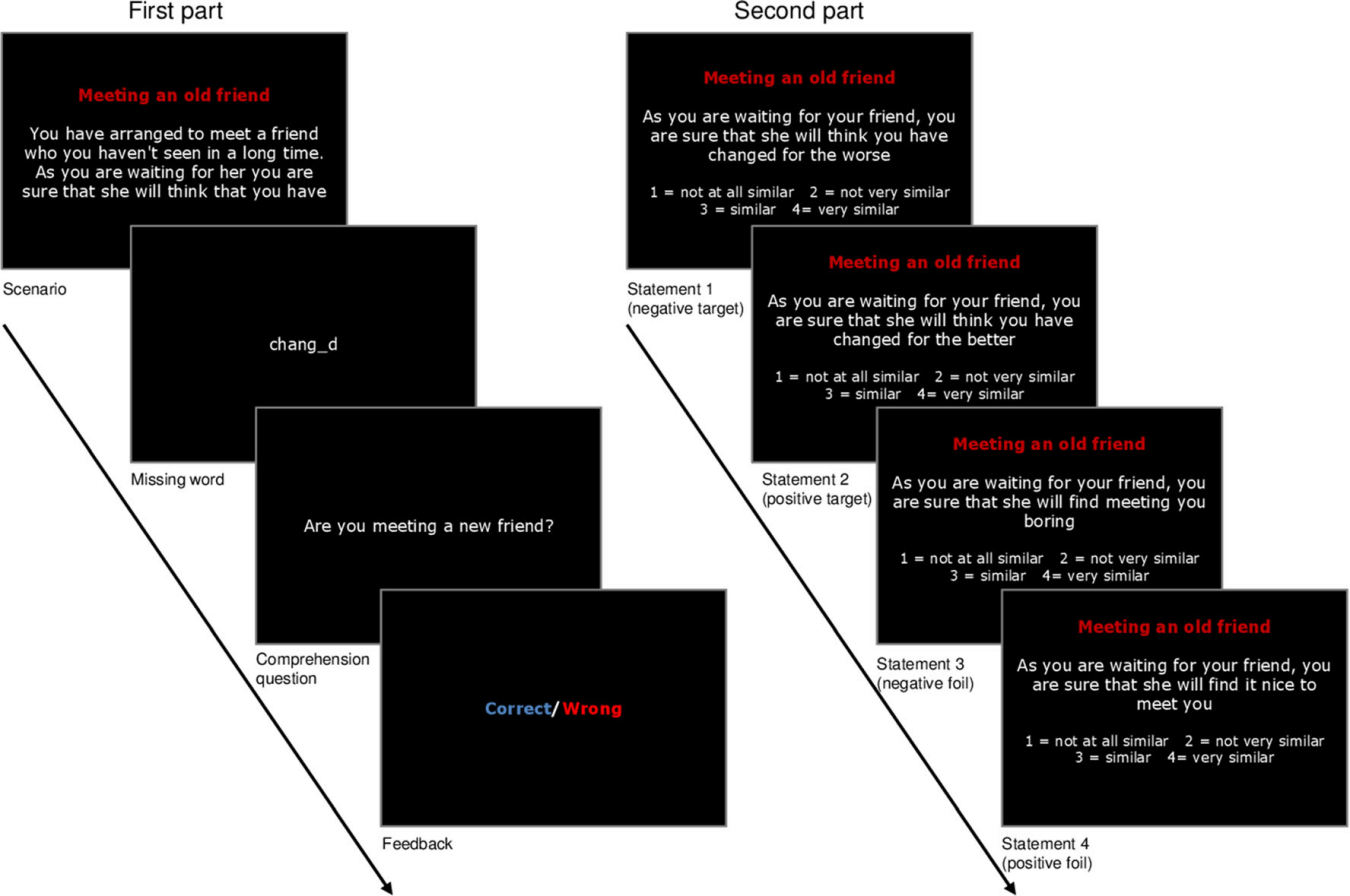
Example scenario from the Ambiguous Scenarios Task (AST; Mathews and Mackintosh 2000)

Stimuli consisted of ten ambiguous scenarios, i.e., descriptions of self-referent situations that could be interpreted either positively or negatively. Stimuli were based on the original stimulus set by Mathews and Mackintosh (2000) which was developed to assess interpretation biases in relation to anxiety. The set was translated and adapted to be age-appropriate (by creating situations related to school, sports, or friends to which the studied age group could relate; Belli and Lau 2014; Klein et al. 2018; Lothmann et al. 2011) and more depression-specific (by including not only social situations that might lead to rejection but also situations targeting low self-esteem and the tendency to overgeneralize/catastrophize potentially negative events, which are typical of depressive thinking). Separate versions for girls and boys were generated (differing mainly in using female or male words when referring to, e.g., friends or classmates). See Fig. 1 for an example scenario (and Sfärlea et al. 2019, Supplement 3, for an English translation of all scenarios).

#### Task Procedure

The trial procedure is depicted in Fig. 1. The experiment was presented using E-Prime 2.0 (Psychology Software Tools Inc 2013). In the first part of the task, each trial started with the title and the description of a situation with one word missing at the end. Participants were instructed to read the description carefully and to imagine they were in that situation. After reading the description, participants pressed the spacebar to reveal a fragment of the missing word. They completed the word by typing in the missing letter. Subsequently, a comprehension question that had to be answered by pressing “J” for Yes and “N” for No was presented, followed by feedback. The word completion and comprehension question were included to ensure that participants read the scenarios carefully.

After the first part, the task continued with a second part in which the title of each scenario was presented with four probe statements. Participants had to rate the similarity of the statements to the original scenario from 1 (“not similar at all”) to 4 (“very similar”). The statements consisted of one valid negative and one valid positive interpretation (targets), as well as one negative statement and one positive statement that were not directly related to the scenario (foils). For each scenario, the four probe statements were presented consecutively in random order.

The ten scenarios were presented in random order in both parts and were preceded by one neutral scenario to familiarize participants with the task.

#### Outcome Variables

An interpretation bias score (IB_AST_) was calculated by subtracting the mean positive target score from the mean negative target score (e.g., de Voogd et al. 2017) so that scores > 0 indicated a negative interpretation bias and scores < 0 indicated a positive interpretation bias. A foil ratio was similarly calculated. Comparing the interpretation bias score and the foil ratio allows analyzing the endorsement of negative versus positive interpretations of ambiguous scenarios (i.e., an interpretation bias, represented by the IB_AST_ score) compared to the tendency to simply endorse non-specific negative versus positive statements (i.e., a negative response bias, represented by the foil ratio; Belli and Lau 2014).

#### Reliability

Split-half reliability of the task was assessed by correlating bias scores based on odd versus even trials (see e.g., Van Bockstaele et al. 2017) and was good (*r* = .66, *p* < .001, Spearman-Brown-corrected reliability: .80).

### Scrambled Sentences Task

A computerized version of the SST (Wenzlaff and Bates 1998; adapted by Everaert et al. 2014) was used to assess the tendency to form negative or positive statements out of ambiguous verbal information. The task was administered during eye-tracking in order to simultaneously assess attention biases (Everaert et al. 2014), but these data are reported elsewhere (Buhl et al. in preparation).

#### Stimuli

The stimuli consisted of 50 scrambled sentences: 30 emotional sentences (e.g., “total I winner a loser am”) and 20 neutral sentences (e.g., “like watching funny I exciting movies”). The emotional sentences were based on the original stimulus set developed by Wenzlaff and Bates (1998) and included, e.g., sentences targeting low mood, low self-worth, and negative thoughts about oneself and the future, which are typical cognitions in depression. The sentences were translated into German (Rohrbacher 2016), extended, and adapted to be easily understandable and relevant to children and adolescents (see Supplement 4 of Sfärlea et al. 2019, for an English translation of the stimuli). All sentences contained six words and had two possible solutions. In emotional trials, one solution was positive (e.g., “I am a total winner”) whereas the other was negative (e.g., “I am a total loser”). In neutral trials both solutions were emotionally neutral. Across the stimulus set, target words (the words in each sentence that accounted for the positive or negative solution) were matched for length and frequency in the German language.^6^ In line with Everaert et al. (2014), word position within each sentence was randomized, with target words not allowed next to each other or in the first or last position and counterbalanced whether the positive or negative target word was presented first.

#### Task Procedure

**Fig. 2:**
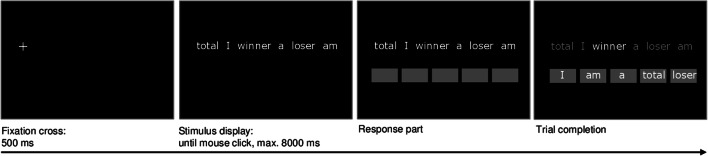
Example of an emotional trial of the Scrambled Sentences Task (SST; Everaert et al. 2014; Wenzlaff and Bates 1998)

The trial procedure is depicted in Fig. 2. The experiment was presented using Experiment Builder 1.10 (SR Research Ltd 2013). Each trial started with a fixation cross presented for 500 ms on the left side of the screen. After that, the stimulus display appeared, consisting of six words in scrambled order presented at the center of the screen on a single line. Participants were instructed to read the words, mentally form a grammatically correct five-word sentence as quickly as possible, and click on the mouse button as soon as they did so to continue to the response part of the trial. The scrambled sentence was presented for a maximum of 8000 ms; if no mouse click occurred during that time the response part was omitted and the next trial began. In the response part, five boxes appeared below the scrambled sentence and participants were required to build the sentence they had mentally formed by ordering the words into the five boxes via mouse click.

Trials were randomly divided into five blocks of ten, each containing six emotional and four neutral trials presented in random order. Before the first block participants completed five practice trials to familiarize themselves with the task.

Similarly to earlier studies (e.g., Everaert et al. 2014; Burnett Heyes et al. 2017) a cognitive load procedure was included to prevent deliberate response strategies. Before each block, a 4-digit number was presented for 5000 ms which had to be memorized and recalled at the end of the block.

#### Data Processing and Outcome Variables

Participants’ responses were rated as correct or incorrect. Trials in which no grammatically correct sentence was built (time-out or incorrect sentence) were excluded from the analysis. Participants with a correct sentence rate of three standard-deviations (*SD*) below the mean were identified as outliers in terms of accuracy and excluded (2 HR children), resulting in a sample of 119 children (as data from only 121 of 122 participants were available for the SST due to technical problems) for analysis of the SST data. In that remaining sample, on average 44.2 correct trials (*SD* = 4.1; 88% of 50 trials) per participant were available (not different between groups, *p* > .1).

The correctly unscrambled emotional sentences were categorized as either positive or negative. An interpretation bias score (IB_SST_) was calculated as the proportion of negatively resolved sentences from the total number of correctly resolved emotional sentences (Everaert et al. 2014).

#### Reliability

Split-half reliability of the SST was calculated analogous to the AST and was excellent (*r* = .89, *p* < .001, Spearman-Brown-corrected reliability: .94).

### Experiment Procedure

Tasks were administered in random order. The course of the experimental session was the same as in Sfärlea et al. (2019; see Supplement 5).

As cognitive models of depression suggest that cognitive vulnerabilities such as negative biases are activated by stressful life events or negative mood (e.g., Disner et al. 2011; Scher et al. 2005), a negative mood induction procedure was administered twice during the experimental session: Participants watched a 2 min scene from the movie *The Lion King* (Hahn et al. 1994) that had successfully induced negative mood in children in earlier studies (von Leupoldt et al. 2007). In our study participants also reported significantly worse mood (assessed using the valence dimension of the 9-point Self-Assessment Mannequin scale; Lang 1980) after watching the movie scene compared to baseline (*t*s ≥ 7.9, *p*s < .001). Details are presented in Supplement 2.

### Data Analysis

Statistical data analysis was conducted with SPSS 25. To assess group differences in demographic and clinical characteristics, interpretation bias scores (IB_AST_ and IB_SST_),^7^ as well as the AST foil ratio, one-way analyses of variance (ANOVAs) and follow-up *t*-tests (Bonferroni-Holm corrected; Holm 1979) were conducted. Correlations were calculated between bias scores and depression and anxiety symptoms to assess relationships between psychopathology and interpretation bias. Furthermore, in order to examine if interpretation bias scores from the two tasks were related, a correlation between IB_AST_ and IB_SST_ scores was computed.

All analyses were repeated excluding the participants that were taking psychotropic medication, as this might influence cognitive biases (e.g., Wells et al. 2014). As the overall pattern of results remained the same, the findings based on the whole sample are reported.

## Results

### Sample Characteristics

Sample characteristics are presented in Table 1. Groups did not differ significantly in gender ratio or IQ but in terms of age: participants in the MD group were significantly older than participants in the HR and LR groups. To examine whether interpretation bias scores were related to age, Pearson’s correlations between age and IB_AST_ as well as IB_SST_ scores were calculated separately for each group: no significant correlations emerged (*r*s ≤ .29, *p*s > .1). As expected, groups also differed in psychopathology with the MD group reporting significantly more symptoms of depression and anxiety than the groups of healthy children (which did not differ from each other, further indicating that the HR group was indeed as psychiatrically healthy as the LR group yet).

**Table 1:** Demographic and clinical characteristics of the sample

	MD	HR	LR			Post-hoc tests
	*n* = 32	*n* = 48	*n* = 42			
Gender m/f	6/26	19/29	17/25	*χ*^*2*^ = 4.7	n.s.	
Age; *M (SD)*	13.4 (1.4)	11.8 (1.7)	12.2 (1.7)	*F*_2,119_ = 9.3	*p* < .001	MD > HR = LR
IQ; *M (SD)*	105.2 (13.6)	109.1 (11.5)	111.7 (10.3)	*F*_2,119_ = 2.8	n.s.	
Depression symptoms; *M (SD)*	31.5 (8.9)	7.8 (5.8)	6.6 (5.3)	*F*_2,118_ = 161.0	*p* < .001	MD > HR = LR
Anxiety symptoms; *M (SD)*	45.1 (8.8)	30.1 (6.4)	28.0 (6.2)	*F*_2,114_ = 56.3	*p* < .001	MD > HR = LR

*MD* Major depression, *HR* high-risk, *LR* low-risk

Depressive symptoms were assessed with the DIKJ (raw values presented) and anxiety was assessed with the STAIC-T. Post-hoc *t*-tests were all significant with *p* ≤ .001

### Ambiguous Scenarios Task

The one-way ANOVA revealed a significant effect of group (*F*_2,119_ = 13.0, *p* < .001, *η*^*2*^ = .18) that was followed up by *t*-tests: the MD group’s IB_AST_ score was significantly more negative than that of the HR group (*t*_48.0_ = 4.1, *p* < .001, *d* = 1.0) and the LR group (*t*_44.8_ = 3.3, *p* = .002, *d* = 0.8), while the HR and LR groups did not differ from each other (*t*_88_ = 1.5, *p* > .1). The IB_AST_ score was significantly > 0 in the MD group (*M* = 0.4, *SD* = 1.0; *t*_31_ = 2.2, *p* = .034), indicating a negative interpretation bias, and significantly < 0 in the HR and LR groups (HR: *M* = −0.4, *SD* = 0.6; LR: *M* = −0.2, *SD* = 0.5; *t*s ≥ 2.9, *p*s ≤ .006), indicating a positive interpretation bias.

**Fig. 3:**
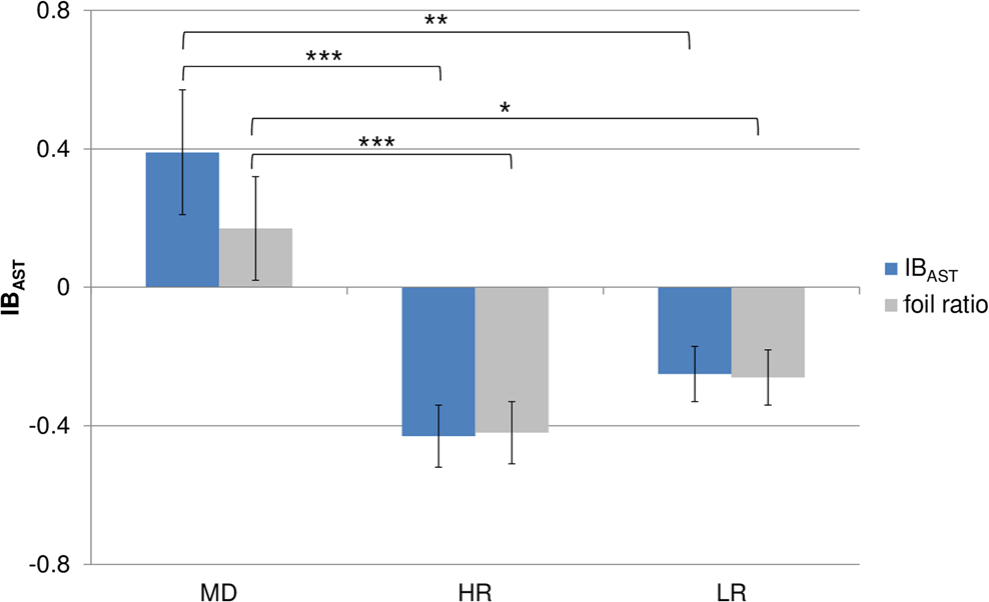
IB_AST_ scores and foil ratios for the three groups. Error bars represent standard errors. Significant group differences are indicated: *** *p* < .001, ** *p* < .01, * *p* < .05

The one-way ANOVA on foil ratios also yielded a significant effect of group (*F*_2,119_ = 8.0, *p* = .001, *η*^*2*^ = .12) with similar results in the post-hoc *t*-tests but smaller effect sizes (MD vs. HR: *t*_78_ = 3.6, *p* = .001, *d* = 0.8; MD vs. LR: *t*_47.9_ = 2.6, *p* = .013, *d* = 0.6; HR vs. LR: *t*_88_ = 1.3, *p* > .1). *T*-tests against zero revealed that while foil ratios in the HR (*M* = −0.4, *SD* = 0.6) and LR groups (*M* = −0.3, *SD* = 0.5) were significantly < 0 (*t*s ≥ 3.3, *p*s ≤ .002), the foil ratio of the MD group (*M* = 0.2, *SD* = 0.9) was not significantly different from zero (*t*_31_ = 1.2, *p* > .1). IB_AST_ scores and foil ratios are presented in Fig. 3.

Furthermore, positive correlations between IB_AST_ scores and depression (*r* = .44, *p* < .001) as well as anxiety symptoms (*r* = .41, *p* < .001) were found. These two correlations did not differ in size (*z* = 0.4, *p* > .1; Lee and Preacher 2013). As the groups differed in both, psychopathology scores as well as IB_AST_ scores, the correlational analyses were repeated within the groups. In the MD group, significant correlations between IB_AST_ scores and depression (*r* = .39, *p* = .026) as well as anxiety symptoms (*r* = .39, *p* = .047) emerged, while in the HR and LR groups no such correlations were apparent (*r*s ≤ .22, *p*s > .1).

### Scrambled Sentences Task

**Fig. 4:**
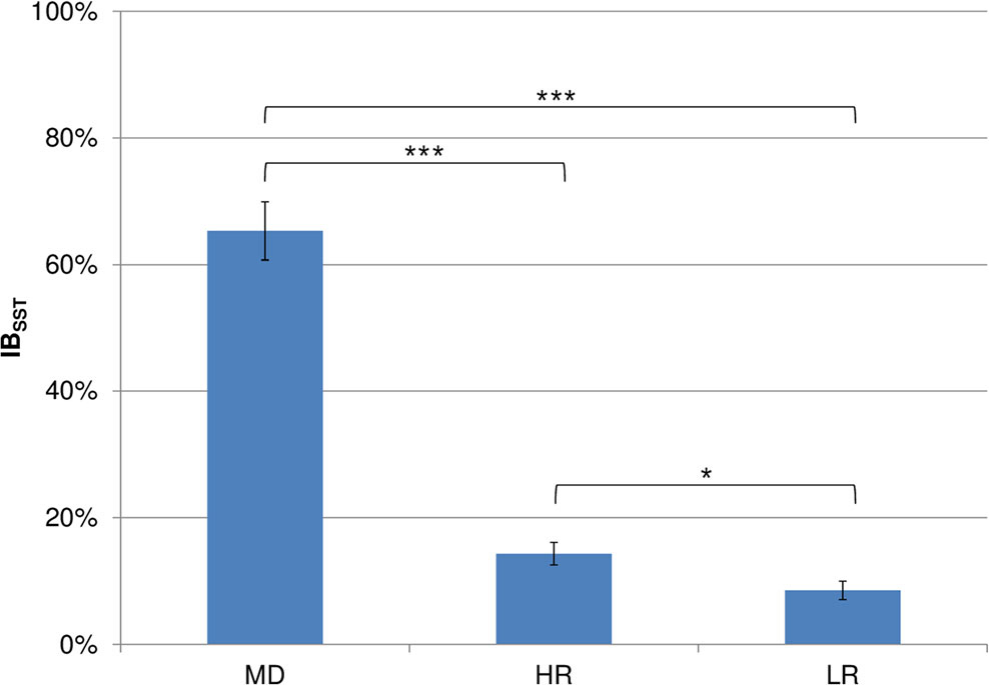
IB_SST_ scores for the three groups. Error bars represent standard errors. Significant group differences are indicated: *** *p* < .001, * *p* < .05

The one-way ANOVA on IB_SST_ scores revealed a significant effect of group (*F*_2,116_ = 129.0, *p* < .001, *η*^*2*^ = .69) that was followed up by *t*-tests: the MD group (*M* = .65, *SD* = .26) had a significantly more negative bias than the HR (*M* = .14, *SD* = .12; *t*_40.7_ = 10.4, *p* < .001, *d* = 2.5) and LR (*M* = .08, *SD* = .09; *t*_37.3_ = 11.8, *p* < .001, *d* = 2.9) groups, and the HR group had a more negative interpretation bias than the LR group (*t*_82.5_ = 2.5, *p* = .014, *d* = 0.6). Results are presented in Fig. 4.

Strong positive correlations of IB_SST_ scores with symptoms of both depression (*r* = .89, *p* < .001) as well as anxiety (*r* = .72, *p* < .001) were found, although the relationship with depressive symptoms was significantly stronger than with anxiety (*z* = 5.7, *p* < .001; Lee and Preacher 2013). When recalculated within groups, correlations of IB_SST_ scores with depressive symptoms were evident in each group (MD: *r* = .70, *p* < .001; HR: *r* = .56, *p* < .001; LR: *r* = .43, *p* = .005) and correlations with anxiety symptoms became apparent in the MD (*r* = .39, *p* = .046) and HR groups (*r* = .48, *p* = .001; LR: *r* = .22, *p* > .1).

### Relationship between AST and SST

A significant positive correlation between IB_AST_ and IB_SST_ scores emerged across groups (*r* = .53, *p* < .001) but within groups this relationship was only found in the MD group (*r* = .56, *p* = .001; HR and LR: *r*s ≤ .18, *p*s > .1).

## Discussion

The present study investigated the role of interpretation biases in youth depression. Two experimental tasks capturing different aspects of interpretation were used to assess interpretation biases in three groups of children and adolescents: currently depressed children and adolescents (MD group), children and adolescents at high risk for depression due to having a parent with a history of depression (HR group), and children and adolescents with a low risk for depression (LR group). Both tasks revealed a more negative interpretation bias in children and adolescents with MD compared to both groups of healthy youth and strong correlations between bias scores and depression and anxiety symptoms (collapsed across groups), while only one task (SST) revealed a more negative interpretation bias in youth at risk for depression compared to low-risk youth (see also Sfärlea et al. 2019).

The first aim of the present study was to test the assumption that children and adolescents with MD show more negative interpretation biases compared to healthy youth. As expected, we found the MD group to draw more negative interpretations of ambiguous scenarios (AST) as well as sentences (SST), i.e., to show more negative interpretation biases, than the two groups of healthy children and adolescents. The effect sizes of the group differences were large, especially for the SST, and comparable to those found with questionnaire measures of interpretation bias (Orchard et al. 2016b). Of note, as we calculated relative bias scores, our results do not elucidate if the more negative interpretation biases in depressed children and adolescents were due to a lack of positive interpretations or an excess of negative interpretations. However, an additional analysis of the AST data with absolute positive and negative scores instead of a relative bias score indicated that group differences in the AST were mainly driven by the MD group being more likely to endorse *negative* interpretations compared to HR and LR groups while no differences were found for positive interpretations (results of this analysis are presented in Supplement 3). It also has to be acknowledged that the foil ratio of the AST was also more negative in the MD group than in the HR and LR groups (although with smaller effect sizes: *d* = 0.6–0.8 vs. *d* = 0.8–2.9). As the foil ratio represents the tendency to endorse non-specific negative statements this suggests that the more negative interpretation bias in the MD group may partly be explained by a more general negative response bias. Our study is the first to focus on comparing interpretation biases in depressed versus non-depressed youth using multiple experimental measures. The results extend those of prior studies that have investigated interpretation biases in depressed adolescents (aged 12–18; Orchard et al. 2016b; and 14–21 years; Micco et al. 2014) to a younger age group. The presence of negative interpretation biases in depressed children and adolescents corroborates the assumption that negative interpretation biases are a characteristic of individuals with depression not only in adults and adolescents but also in 9–14 year old youth and provides empirical support that cognitive theories of depression (e.g., Disner et al. 2011) apply to this group as well. However, as it remains unclear how interpretation biases emerge across childhood and adolescence, future studies may compare interpretation biases between different age groups, e.g., children vs. adolescents, or investigate interpretation biases longitudinally across childhood and adolescence.

The bias score was strongly positively related to depressive symptoms in the full sample, replicating previous results in youth with depression (Micco et al. 2014) or elevated symptoms of depression (de Voogd et al. 2017) as well as unselected samples of adolescents (e.g., Klein et al. 2018; Orchard et al. 2016a). However, when correlations were calculated separately within each group, consistent correlations with depressive symptoms were found only for interpretation bias as assessed with the SST, while the interpretation bias assessed with the AST only correlated with depressive symptoms within the MD group, probably due to lower values and/or less variance of depression, anxiety, and IB_AST_ scores in the HR and LR groups. Similar relationships were found for anxiety symptoms, which is not surprising considering the well-established association of anxiety and interpretation biases in children and adolescents (Stuijfzand et al. 2018). However, a comparison of the correlation coefficients indicated that for the interpretation bias score as assessed with the SST, the association with depressive symptoms was significantly stronger than the association with anxiety symptoms, suggesting at least partial specificity. For the interpretation bias score as assessed with the AST, on the other hand, correlations with symptom scores did not differ.

The second aim of the study was to determine the extent to which interpretation biases are more pronounced in currently depressed youth compared to at-risk youth. In line with our expectations and previous studies (Dearing and Gotlib 2009), children and adolescents at high risk for depression showed a more negative interpretation bias compared to children and adolescents at low risk for depression (see also Sfärlea et al. 2019). However, only the interpretation bias as assessed with the SST (not the AST) was more negative in the HR group than in the LR group and it was much less pronounced than in the MD group. This is the first time interpretation biases are compared between currently depressed children and adolescents and children and adolescents with a high or low risk for depression. The results indicate that while being to some extent already present in at-risk populations,^8^ negative interpretation biases are strongly exacerbated in currently depressed children and adolescents.

The two tasks assessing interpretation biases yielded divergent results: the AST differentiated only between depressed and non-depressed children and adolescents and was related to depressive symptoms only within the MD group, while the SST also differentiated between high- and low-risk youth and was associated to depressive symptoms within all groups. Moreover, interpretation bias scores from the two tasks were only related within the MD group. Based also on our previous results (Sfärlea et al. 2019), we suppose that the AST and the SST capture different aspects of interpretation (an issue which Everaert et al. 2017, pointed out as especially important to investigate): the SST is more cognitively demanding due to the time constraint and the cognitive load procedure, so less resources are left for volitional control and deliberate response strategies. Therefore, the SST may capture a more automatic (in terms of quick and effortless processing that occurs unintentionally and uncontrollably; cf. Beevers 2005; Teachman et al. 2012) and implicit aspect of interpretation. The AST, on the other hand, allows more reflection on one’s answers and might therefore be more susceptible to distorted responding, similarly to self-report measures (e.g., Gotlib and Joormann 2010). Hence, the AST presumably measures a more conscious and explicit aspect of interpretation (see Sfärlea et al. 2019, for more details). According to this assumption, our results suggest that an implicit interpretation bias can already be found in at-risk youth before onset of a depressive disorder and thus might act as a cognitive vulnerability or risk factor contributing to the development of depression (as suggested by theoretical models; e.g., Disner et al. 2011). The explicit interpretation bias, on the other hand, was only found in the currently depressed group, indicating that this type of bias may arise as a consequence of depressive symptomatology. The finding that these two aspects of interpretation operate differently with respect to the question of being present already in youth at risk for depression or only in currently depressed children and adolescents contributes to a more comprehensive and differentiated understanding of interpretation biases in youth depression. However, the cross-sectional design of the study does not allow any conclusions about time course or causality: we cannot determine the predictive value of interpretation biases for prospectively predicting the onset of an episode of MD, i.e., whether the more negative interpretation bias in the HR group compared to the LR group indeed acts as a risk factor for the development of MD. Likewise, we cannot conclude if the more negative interpretation biases we found in the MD group compared to the HR group are consequences of the depressive disorder or had already characterized those individuals that developed MD before disorder onset. Longitudinal research is needed to address these important questions as well as to investigate what role negative interpretation biases play in the maintenance of depressive symptoms.

### Clinical Implications

We found strong negative interpretation biases in children and adolescents with MD on explicit as well as implicit levels. This suggests that therapeutic attempts to modify these biases in depressed youth might be more efficient if they address interpretation biases not only explicitly via Cognitive Behavioral Therapy (e.g., J. S. Beck 2011) but also implicitly, for example via Cognitive Bias Modification interventions that have been shown to successfully modify interpretation biases not only in healthy (Lothmann et al. 2011) but also in depressed adolescents (LeMoult et al. 2018; Micco et al. 2014).

The presence of negative implicit interpretation biases also in youth at high risk for depression, on the other hand, indicates that this kind of interpretation bias might also be a target for preventive approaches trying to reduce the impact of cognitive vulnerabilities in children of depressed parents. Modifying cognitive processes using implicit methods might enhance the efficacy of prevention programs in this high-risk group, whose effects are rather small and short-term (Loechner et al. 2018). However, as some studies implementing Cognitive Bias Modification interventions for interpretation bias reported that those lacked transfer effects (e.g., LeMoult et al. 2018; Yiend et al. 2014), these interventions clearly need to be refined and improved before representing useful therapeutic tools for treatment and prevention of depressive disorders. Moreover, as any intervention intended for younger age groups, Cognitive Bias Modification interventions for children and adolescents need to be age-adapted, e.g., by using picture-based instead of text-based stimuli for younger children.

Furthermore, as the two measures of interpretation bias presumably capture different aspects of interpretation, the AST and the SST could be useful tools for assessing the extent to which existing interventions are able to change interpretation biases in children and adolescents with MD separately on conscious as well as automatic levels.

### Strengths

The present study makes a significant contribution to our knowledge of the role of interpretation biases in youth depression holding several methodological strengths.

Two different tasks were administered to experimentally assess interpretation biases. The reliability of the tasks was determined and turned out to be at least good for both measures (corresponding to e.g., Micco et al. 2014; Novović et al. 2014). Furthermore, the correlations between bias scores and depressive symptoms underline the construct validity of the measures as indicators of depressive processing.

Moreover, not only did all participants included in the study undergo extensive diagnostic assessment, psychopathology was also carefully assessed in one (HR group) or both (LR group) of their parents via a diagnostic interview instead of relying on self-report of mental disorder history only.

### Limitations

One limitation of the present study is that the three groups investigated differed in age with participants in the MD group being significantly older than participants in the HR and LR groups. This probably results from the prevalence of depression being rather low in childhood and rising substantially with puberty (Thapar et al. 2012) and therefore the majority of the participants in the MD group being 12 to 14 years old. However, as age was not related to bias scores, it is unlikely that the age difference accounts for the group differences we found.

Another limitation results from nearly half of the participants in the MD group having a comorbid anxiety disorder. Also, not only depressive but also anxiety symptoms were related to interpretation biases, which was to be expected considering that the stimuli used in the tasks – even though adapted to our study population – were not entirely depression-specific due to the symptom overlap between depression and particular anxiety disorders like social anxiety disorder or generalized anxiety disorder. Therefore, it cannot be ruled out that comorbid psychopathology contributed to our results. However, the association with depressive symptoms was stronger than the association with anxiety symptoms (for the SST, which is the more depression-specific measure), suggesting at least partial specificity.

Furthermore, it remains unknown if group differences in interpretation bias, particularly the difference between HR and LR groups in the SST, can also be observed during baseline mood and without the cognitive load, as interpretation biases were only assessed following a negative mood induction and the SST was not applied without the cognitive load procedure. These possibilities should be addressed by future studies as they have important implications for cognitive models of depression.

Finally, since most participants in the MD group were recruited at a Department of Child and Adolescent Psychiatry or through licensed outpatient psychotherapists, it is likely that most of them were receiving some form of psychotherapy at the time of their participation (unfortunately, this was not assessed systematically). Since psychotherapy, particularly Cognitive Behavioral Therapy, targets negative interpretation biases, our effect sizes might be underestimates of the effect sizes in untreated youth depression. Furthermore, since a considerable proportion of the participants in the HR group were recruited through a study evaluating a family-based prevention program for children of parents with a history of depression (Platt et al. 2014), our HR participants might have been less vulnerable to depression than the average offspring of depressed parents (see Sfärlea et al. 2019, for a more detailed discussion).^9^ In summary, our MD and HR samples might not be entirely representative and group differences might be underestimated in our study.

## Conclusion

The present study provides evidence for the presence of explicit as well as implicit negative interpretation biases in children and adolescents with MD and implicit interpretation biases in children and adolescents at risk for depression. Pending replication in longitudinal studies, this suggests that implicit interpretation biases might represent cognitive vulnerabilities for depression while explicit interpretation biases seem to arise as a consequence of depression. The results have important clinical implications for the improvement of interventions to prevent and treat youth depression.

## Electronic supplementary material

ESM 1 (DOCX 40 kb)

## Footnotes

1 In addition to the AST and the SST that are presented here, a short, picture-based task (resembling that used by Haller et al. 2016) was piloted. However, the validity of this task was limited in our study (see Supplement 1).

2 Altogether, 126 children and adolescents were tested. Two participants were excluded due to bad compliance and two because of severe reading difficulties (as both interpretation bias tasks are based on reading). The sample size was based on an a priori power analysis (α error probability = .05, power = .8, one-tailed) for the comparison of HR and LR groups (as a smaller effect size was expected for this effect than for the comparisons with the MD group). An effect size around *d* = 0.6 (corresponding to Dearing and Gotlib 2009) was expected, therefore a sample of at least *n* = 36 per group was aimed for.

3 Two of the participants in the MD group scored just below 85. However, the substandard IQ did not correspond to the clinical impression and was most likely due to a lack of compliance and inability to concentrate on that particular day so those participants were still included.

4 DSM-IV criteria were used as the diagnostic interviews for DSM-V were not available in German by the beginning of data collection.

5 One girl met criteria for enuresis in the past. However, as she did not report symptoms of any other mental disorder she was included nonetheless.

6 Positive target words: Word length *M* = 7.3 (*SD* = 2.6) characters, word frequency (category according to http://wortschatz.uni-leipzig.de/de) *M* = 10.3 (*SD* = 2.9); Negative target words: word length *M* = 7.4 (*SD* = 2.6) characters, word frequency *M* = 10.3 (*SD* = 4.0); *t*s < 1 in paired *t*-tests.

7 Relative bias score were used since significantly larger effect sizes were reported for studies computing relative bias scores compared to studies computing absolute positive or negative bias scores (Everaert et al. 2017). In addition, relative bias scores allow to examine if bias scores from different tasks are related to each other. An alternative analysis of the AST with absolute positive and negative values can be found in Supplement 3.

8 Note that parental history of depression is not the only risk factor for depression. Psychosocial factors like exposure to stressful life events (e.g., bereavement) or chronic adversity (e.g., maltreatment, bullying) also put children and adolescents at risk for developing depression (Thapar et al. 2012). It remains unclear if our results are specific for children of depressed parents or apply to other risk groups as well.

9 Analyses excluding the 11 children and adolescents that had already participated in the prevention program before taking part in the present study indeed yielded a larger effect size (*d* = 0.7) for the difference between HR and LR children in the SST.
