# Supplementary material for: “I Am a Total…Loser” – The Role of Interpretation Biases in Youth Depression
Source: J Abnorm Child Psychol. 2020 Jul 11;48(10):1337–50. doi: 10.1007/s10802-020-00670-3 (PMC7445197; doi:10.1007/s10802-020-00670-3)
Supplement: Supplementary file 1 — (DOCX 40 kb) [file 10802_2020_670_MOESM1_ESM.docx]

**SUPPLEMENTARY MATERIAL**

**Supplement 1: Picture-based task**

**Method**

An explorative, picture-based task (PBT) resembling that used in the study of Haller, Raeder, Scerif, Kadosh, and Lau (2016) was additionally applied to assess interpretation bias.

Ten pictures of social scenes (similar to those used by Haller et al, 2016; Haller, Doherty, Duta, Kadosh, Lau, & Scerif, 2017) associated with possible peer evaluation served as stimuli. In each picture a person (female in eight and male in two of the pictures) was depicted from behind. Participants were instructed to imagine they were that person and were in that situation. Pictures were presented in random order for five seconds, each followed by three statements. The statements consisted of a positive self-related interpretation of the situation (e.g., “They want me to take a picture with them”), one negative self-related interpretation (e.g., “They do not want me to be in the picture”) and one interpretation unrelated to one’s presence (e.g., “They are having fun taking pictures”). The three statements were presented consecutively and participants were asked to rate on a Likert scale from 1 (“very unlikely”) to 7 (“very likely”) how likely they would be to think that way if they were in that situation. After that, all three statements were presented simultaneously and participants were asked to select which statement represented the picture the best. The task was presented using E-Prime 2.0 software (Psychology Software Tools, Inc., 2013) and took approximately five minutes to complete.

In accordance with Haller et al. (2016), the mean likelihood rating was calculated for each interpretation type and subjected to a repeated-measures analysis of variance (ANOVA) with within-subjects factor InterpretationType (3: positive, negative, unrelated) and between-subjects factor Group (3: MD, HR, LR).

In addition, an interpretation bias score (IB_PBT_) was calculated by subtracting the mean likelihood rating for positive interpretations from the mean likelihood rating for negative interpretations so that scores > 0 indicate a negative interpretation bias and scores < 0 indicate a positive interpretation bias (analogous to the IB_AST_ score). A one-way ANOVA was calculated to assess group differences the IB_PBT_ and correlations between IB_PBT_ and depression as well as anxiety scores were computed.

**Results**

The ANOVA yielded a significant main effect of InterpretationType (*F*_1.4,165.7_ = 32.3, *p* < .001, *η_p_²* = .22) resulting from positive interpretations being rated as less likely to occur than negative and unrelated interpretations (see Supplementary Figure 1). The main effect of Group (*F*_2,118_ = 1.5, *p* > .1) as well as InterpretationType × Group (*F*_1.4,165.7_ = 2.2, *p* = .091) were non-significant.

**Supplementary Figure 1:** Mean likelihood ratings for the three interpretation types. Error bars represent standard deviations.

IB_PBT_ scores were significantly > 0 (MD: *M* = 1.5, *SD* = 1.7; HR: *M* = 0.6, *SD* = 1.9; LR: *M* = 0.9, *SD* = 1.6; *t*s ≥ 2.4, *p*s ≤ .021), indicating a negative bias in all groups. The ANOVA on IB_PBT_ scores revealed no significant effect of Group (*F*_2,118_ = 2.6, *p* = .076). Positive correlations between IB_PBT_ score and depressive symptoms (*r* = .29, *p* = .002) as well as anxiety symptoms (*r* = .23, *p* = .013) were found. Exploratively, the correlations were recalculated within the groups, revealing a significant correlation only between IB_PBT_ and depressive symptoms within the MD group (*r* = .40, *p* = .023), while all other correlations were non-significant (|*r*s| ≤ .26, *p*s > .1).

**Differences between our task and that of Haller et al. (2016):**

Our task differed from that of Haller et al. (2016) in some important aspects which leads to the assumption that the task is a less valid measure of interpretation bias in our study. First and most importantly, the age range of participants differed: while Haller et al. (2016) studied adolescents aged 14-17 years, our sample was 9-14 years old. Therefore it is likely that the stimuli were not age-appropriate for our younger sample. Second, Haller et al. (2016) superimposed a picture of the participant onto the pictures (so that the person depicted from behind was the participant him- or herself) to create the illusion of the participant being an active partaker within the scene. This might render the pictures more self-relevant. Third, a mental imagery task was carried out before the completion of the PBT in the study of Haller et al. (2016), again possibly rendering the pictures more self-relevant. Forth, the task was designed to study the impact of social anxiety on the interpretation of social situations; it might be less suitable to assess interpretation biases related to depression.

**References**

Haller, S. P., Raeder, S. M., Scerif, G., Kadosh, K. C., & Lau, J. Y. (2016). Measuring online interpretations and attributions of social situations: Links with adolescent social anxiety. *Journal of Behavior Therapy and Experimental Psychiatry, 50*, 250-256. doi: 10.1016/j.jbtep.2015.09.009

Haller, S. P., Doherty, B. R., Duta, M., Kadosh, K. C., Lau, J. Y., & Scerif, G. (2017). Attention allocation and social worries predict interpretations of peer-related social cues in adolescents. *Developmental Cognitive Neuroscience*, *25*, 105-112. doi: [10.1016/j.dcn.2017.03.004](https://doi.org/10.1016/j.dcn.2017.03.004)

Psychology Software Tools. (2013). E-Prime 2.0 [Computer software]. Sharpsburg, PA: Author.

**Supplement 2: Results of the mood induction**

Mood was assessed five times during the experimental session (see Sfärlea et al., 2019, Supplement 5) using the valence dimension of the 9-point Self-Assessment Mannequin scale (Lang, 1980). In addition to calculating *t*-tests comparing the baseline mood (mood before first mood induction: Time 1) with mood after the mood inductions (Time 2 and Time 3) across groups (results presented in the main text), we also analysed mood using a Time (5) × Group (3) ANOVA to investigate if the mood induction procedure had the same impact on mood in all three groups.

The ANOVA yielded a significant main effect of Time (*F*_2.1,251.3_ = 56.1, *p* < .001, *η_p_²* = .32), a significant main effect of Group (*F*_2,118_ = 59.1, *p* < .001, *η_p_²* = .50), resulting from the MD group reporting more negative mood than the HR (*t*_46.9_ = 8.2, *p* < .001) and LR groups (*t*_44.8_ = 8.4, *p* < .001), as well as a Time × Group interaction (*F*_2.1,251.3_ = 9.3, *p* < .001, *η_p_²* = .14). To follow-up the interaction, one-way ANOVAs with the factor Time were performed within each group resulting in significant effects within the HR and LR groups (HR: *F*_2.6,121.7_ = 44.0, *p* < .001, *η_p_²* = .48; LR: *F*_1.6,64.5_ = 33.8, *p* < .001, *η_p_²* = .45) but not the MD group (*F*_2.4,70.7_ = 1.2, *p* > .1). This indicated that, in fact, the mood induction only influenced the mood of the non-depressed participants while the mood of participants with MD was more negative before the mood induction and remained so throughout the experimental session (see Supplementary Figure 2).

**Supplementary Figure 2:** Mean mood ratings in each of the five mood assessments. Error bars represent standard deviations.

We further conducted post-hoc *t*-tests comparing baseline mood (mood before first mood induction: Time 1) with the other mood assessments in the HR and LR groups. These analyses revealed that both HR as well as LR participants rated their mood as worse after watching the sad movie scene (Time 2 and Time 3; *t*s ≥ 5.4; *p*s < .001) but only HR participants rated their mood better after watching the pleasant movie scene (Time 5; *t*_47_ = 3.5; *p* = .001) compared to baseline.

**References**

Hahn, D. (Producer), Allers, R., & Minkoff, R. (Directors). (1994). *The Lion King* [Motion Picture]. USA: Walt Disney.

Lang, P. J. (1980). Behavioral treatment and bio-behavioral assessment: Computer applications. In J. B. Sidowski, J. H. Johnson & T. A. Williams (Eds.), *Technology in Mental Health Care Delivery Systems* (pp. 119-137). Norwood: Ablex.

Sfärlea, A., Löchner, J., Neumüller, J., Asperud Thomsen, L., Starman, K., Salemink, E., Schulte-Körne, G., & Platt, B. (2019). Passing on the half-empty glass: A transgenerational study of interpretation biases in children at risk for depression and their parents with depression. *Journal of Abnormal Psychology*, *128*, 151-161.

**Supplement 3: Alternative analysis of the AST**

An alternative way to analyse the AST with absolute positive and negative scores instead of relative scores is to calculate a 2-way ANOVA with the within-subjects factor Valence (2: mean negative vs. positive target scores) and the between-subjects factor Group (3) and corresponding post-hoc tests.

This ANOVA yielded no main effects of Valence (*F*_1,119_ = 2.0, *p* > .1, *η_p_²* = .02) or Group (*F*_2,119_ = 2.1, *p* > .1, *η_p_²* = .03) but a significant Valence × Group interaction (*F*_2,119_ = 13.0, *p* < .001, *η_p_²* = .18). To follow up this interaction, one-way ANOVAs with the between-subjects factor Group were performed separately for mean negative and positive target scores. For negative target scores, this analysis yielded a significant effect of Group (*F*_2,119_ = 13.3, *p* < .001, *η²* = .18) with post-hoc *t*-tests indicating that the MD group was more likely to endorse negative interpretations than the HR (*t*_53.5_ = 4.4, *p* < .001, *d* = 1.0) and LR groups (*t*_48.2_ = 3.4, *p* = .001, *d* = 0.8) while the HR and LR groups did not differ from each other (*t*_88_ = 1.5, *p* > .1, *d* = 0.3). For positive bias scores, the effect of Group was non-significant (*F*_2,119_ = 3.1, *p* = .050, *η²* = .05). Supplementary Figure 3 illustrates the absolute values for endorsement of negative and positive targets.

**Supplementary Figure 3:** Mean absolute values for the endorsement of negative and positive targets. Error bars represent standard deviations.

These results indicate that the group differences in the AST were mainly driven by difference in the endorsement of *negative* interpretations, which the MD group was more likely to endorse than the HR and LR groups. Descriptively, the MD group was also less likely to endorse positive interpretations than the HR and LR groups, but differences for positive interpretations were not significant.
